# Antigenic and Biological Characterization of ORF2–6 Variants at Early Times Following PRRSV Infection

**DOI:** 10.3390/v9050113

**Published:** 2017-05-16

**Authors:** Alyssa B. Evans, Hyelee Loyd, Jenelle R. Dunkelberger, Sarah van Tol, Marcus J. Bolton, Karin S. Dorman, Jack C. M. Dekkers, Susan Carpenter

**Affiliations:** 1 Department of Animal Science, Iowa State University, Ames, IA 50011, USA; alyssa.ben.evans@gmail.com (A.B.E.); heri1008@iastate.edu (H.L.); jenelled@iastate.edu (J.R.D.); savantol@utmb.edu (S.v.T.); mjohnbolton@gmail.com (M.J.B.); jdekkers@iastate.edu (J.C.M.D.); 2 Departments of Statistics and Genetics, Development and Cell Biology, Iowa State University, Ames, IA 50011, USA; kdorman@iastate.edu

**Keywords:** PRRSV, genetic diversity, antigenic variation, replication phenotype, neutralization

## Abstract

Genetic diversity of porcine reproductive and respiratory syndrome virus (PRRSV) challenges efforts to develop effective and broadly acting vaccines. Although genetic variation in PRRSV has been extensively documented, the effects of this variation on virus phenotype are less well understood. In the present study, PRRSV open reading frame (ORF)2–6 variants predominant during the first six weeks following experimental infection were characterized for antigenic and replication phenotype. There was limited genetic variation during these early times after infection; however, distinct ORF2–6 haplotypes that differed from the NVSL97-7895 inoculum were identified in each of the five pigs examined. Chimeric viruses containing all or part of predominant ORF2–6 haplotypes were constructed and tested in virus neutralization and in vitro replication assays. In two pigs, genetic variation in ORF2–6 resulted in increased resistance to neutralization by autologous sera. Mapping studies indicated that variation in either ORF2–4 or ORF5–6 could confer increased neutralization resistance, but there was no single amino acid substitution that was predictive of neutralization phenotype. Detailed analyses of the early steps in PRRSV replication in the presence and absence of neutralizing antibody revealed both significant inhibition of virion attachment and, independently, a significant delay in the appearance of newly synthesized viral RNA. In all pigs, genetic variation in ORF2–6 also resulted in significant reduction in infectivity on MARC-145 cells, suggesting variation in ORF2–6 may also be important for virus replication in vivo. Together, these data reveal that variation appearing early after infection, though limited, alters important virus phenotypes and contributes to antigenic and biologic diversity of PRRSV.

## 1. Introduction

Porcine reproductive and respiratory syndrome virus (PRRSV) is a 15-kb positive-stranded RNA virus belonging to the family *Arteriviridae*, which emerged simultaneously in the United States and Europe in the late 1980s [1,2]. PRRSV is currently classified into two major genotypes that share clinical disease features but differ genetically and antigenically. Type I genotypes are mainly European strains, while Type II genotypes are found primarily in North America and Asia. PRRSV causes respiratory signs in growing pigs and spontaneous abortions in pregnant sows [3], with annual economic losses estimated to be $664 million in the United States alone [4]. Several vaccines have been developed against PRRSV; however, their effectiveness is limited by genetic heterogeneity among field isolates as well as by the continual emergence of antigenic and phenotypic variants [5]. 

Genetic variation can alter susceptibility to neutralizing antibody through antigenic variation in neutralizing epitopes and/or changes in *N*-linked glycosylation sites that mask, or shield, neutralizing epitopes [6,7,8,9]. Neutralizing epitopes have been identified in the major and minor PRRSV envelope proteins, which are encoded by open reading frame (ORF)2–6 [10,11]. ORF5 and ORF6 encode the major envelope proteins GP5 and M, respectively, which interact to form a heterodimer on the virion surface [11] that is believed to be important for attachment [12,13,14,15]. GP5 is highly variable and has long been thought to be the major target of neutralizing antibody [16,17,18,19,20]. Two regions of GP5 have been found to be important for neutralization [16,18,21], and recent studies suggest the neutralization epitope(s) is conformational, rather than linear [20,22,23]. A single tyrosine residue in the highly-conserved M protein was shown to mediate resistance to broadly neutralizing antibody [24]. ORF2–4 encode the minor envelope glycoproteins GP2, GP3, and GP4, respectively, which form an oligomer on the virion surface that interacts with the CD163 receptor [25,26,27,28,29]. Neutralizing antibodies are reported to target GP3 and M in Type II isolates, and GP3, GP4 and M in Type I isolates [8,30,31,32]. Nested within ORF2 and ORF5 are ORF2b and ORF5a, which encode the E and the 5a proteins, respectively [33,34]. Both proteins are minor components of the virion, but are not thought to be targets of neutralizing antibody [34,35]. 

Although neutralizing antibody has been shown to be important for PRRSV clearance [23,36,37,38], the mechanism(s) by which neutralizing antibody acts to inhibit productive virus replication is not known. PRRSV appears to utilize a variety of host cell receptors and viral glycoproteins for attachment, entry, and uncoating [15,39]. The primary site of PRRSV replication in vivo is differentiated porcine alveolar macrophages (PAM), although the virus can be propagated in vitro in the monkey kidney cell line MARC-145. Infection is initiated by low-affinity attachment to heparan sulfate, which is on the surface of both PAM and MARC-145. This interaction, which is mediated by the M protein in the GP5-M heterodimer [12,14], is neither sufficient nor necessary for productive infection. CD169, also referred to as sialoadhesion, facilitates internalization of the virus in PAM, but not in MARC-145 cells, which lack CD169 [14,40,41]. PRRSV is able to establish a productive infection in CD169-knockout pigs [42], demonstrating that CD169 is also not required for virus replication in vivo. In contrast, the scavenger receptor CD163 is absolutely required for PRRSV replication in vitro and in vivo [42,43], and is now recognized as the primary PRRSV receptor. CD163 is expressed in both the endosome and on the cell surface [44], interacts with GP2 and GP4 [28], and is thought to play an essential role in the process of uncoating [45,46]. Most recently, non-muscle myosin heavy chain protein 9 (MYH9) was reported to interact with GP5 and play an essential role at an early step in PRRSV entry [47]. The variety of viral glycoproteins reported to play key roles during the initial stages of PRRSV suggests that, similar to flaviviruses [48], neutralizing antibody may target multiple viral glycoproteins and act at multiple steps during the early stages of PRRSV replication. 

Genetic variation in PRRSV can also lead to changes in biological phenotypes associated with replication and/or virulence, but few studies directly link genotype and phenotype. Genetic variation in both structural and non-structural proteins occurs during sequential passage of PRRSV in vitro, with resultant changes in virulence, immunogenicity, and rate of replication in vivo [49,50]. Increased knowledge as to how specific genetic changes alter virus phenotype can lead to a better understanding of the factors that shape variant selection in vivo. Towards that end, sera samples from five experimentally infected pigs collected at early times after infection were used to characterize ORF2–6 variation and determine the effects of variation on the antigenic and replication phenotype of PRRSV. Limited genetic variation was observed during the first six weeks after infection. However, predominant ORF2–6 haplotypes were identified in each pig which, using reverse genetics, were found to vary in antigenic and/or replication phenotype. These studies indicate that genetic variation arises early after infection and, though limited, alters important virus phenotypes that contribute to antigenic and biologic diversity of PRRSV. 

## 2. Materials and Methods

### 2.1. Cells, Virus, and Pigs

MARC-145 cells used for virus passage and infection assays were maintained in high glucose (4500 mg/L) Dulbecco’s Modified Eagle’s Medium (DMEM) (Sigma-Aldrich Co., LLC., St. Louis, MO, USA) supplemented with 10% FBS, 100 U/mL penicillin, 100 μg/mL streptomycin, and 2 mM l-glutamine. MARC-145 cells used for electroporation were grown in low glucose (1000 mg/L) DMEM (Thermo Fisher Scientific, Waltham, MA, USA) supplemented with 10% FBS, 700 mg/L sodium bicarbonate, 100 U/mL penicillin, and 100 μg/mL streptomycin.

**Figure 1:**
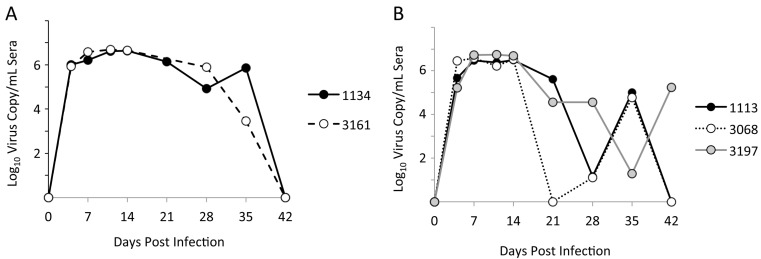
Viremia profiles in porcine reproductive and respiratory syndrome virus (PRRSV)-infected pigs: (**A**) Two pigs with prolonged viremia, as indicated by sustained virus loads >10^3^ copies/mL sera through 35 days post-infection (dpi). (**B**) Three pigs with rebound viremia, indicated by an initial reduction of viremia, followed by greater than 100-fold increase in viremia.

The inoculum virus, NVSL97-7895 (GenBank accession AY545985), was kindly provided by the PRRS Host Genetics Consortium (PHGC). Chimeric viruses were generated in the pFL12 infectious molecular clone backbone, which contains the genome consensus of the NVSL97-7895 inoculum virus [51]. The experimental infections and virus load data were previously described [52,53]. Sera samples from five pigs experimentally infected with NVSL97-7895 were obtained from the PHGC. Two of the pigs had maintained high levels of viremia throughout 35 days post-infection (dpi) (Figure 1A), and three pigs initially cleared the virus, but experienced a rebound in viremia by 42 dpi (Figure 1B).

### 2.2. Cloning and Sequencing PRRSV Variants

Viral RNA was isolated from the NVSL97-7895 inoculum and sera collected from experimentally infected pigs at seven dpi and at a high-viremic late dpi (Table 1) using the QIAamp Viral RNA Mini Kit according to manufacturer’s instructions (Qiagen, Hilden, Germany). Viral RNA was converted to cDNA via random hexamer primers using the Superscript III first strand synthesis kit according to manufacturer’s protocol (Invitrogen, Carlsbad, CA, USA). Approximately 3 kb from PRRSV ORF2–6 was amplified using PRRSV specific primers and high fidelity platinum Taq polymerase (Invitrogen). ORF2–6 was amplified with forward primer 5′ACCAGGTACCGGCCTGAATTGAAATGAAA and reverse primer 5′GGTTGAATTCGGTCAAGCATCTCCCCAAC. Four separate polymerase chain reactions (PCR) from each sample were pooled, purified, TA-cloned into pGEM-T Easy vectors (Promega, Madison, WI, USA) and transformed in Stbl2 *Escherichia coli* cells. Individual colonies were screened for the correct insert size, and positive clones were Sanger sequenced at the Iowa State University DNA Facility (Ames, IA, USA). The sequences were assembled using Phred and Phrap algorithms in MacVector. ORF2–6 was then separated into the individual genes (E, GP2, GP3, GP4, GP5, ORF5a, and M), and each gene was translated to the amino acid sequence. The nucleotide sequences were compared via multiple sequence alignment (ClustalW, MacVector) to determine average pairwise identity and generate consensus sequences. 

To visualize the variation in the sequences, we used the phyclust package (http://cran.r-project.org/web/packages/phyclust/, version 0.1-15) function plotdots, grouping sequences by pig and dpi. Nucleotide changes at each position relative to the consensus nucleotide in the inoculum are shown as colored dots, making it easy to visually identify both single nucleotide variants (SNVs) that appeared with high frequency as well as coordinated changes in variant distributions across multiple pigs.

### 2.3. Identification of Viral Haplotypes

Multiple sequence alignments of the late dpi sample from each individual pig were used to construct viral haplotypes based on the late day virus of each pig. Due to the high mutation rate of the virus, unique single nucleotide changes occurred within most virus clones. Therefore, each haplotype included sites with a minor variant frequency of ≥25% as well as consensus changes from the inoculum that were shared between haplotypes within each individual pig. Each unique combination of nucleotides across the variable sites within a single pig’s virus sample was designated a haplotype. The number of clones within a pig containing each unique haplotype was used to calculate the frequency of that haplotype. The haplotype within each pig present at the highest frequency was designated Haplotype A, and the haplotype present at the second highest frequency was designated Haplotype B. In the case where there was only a single, consensus haplotype, that haplotype is designated Haplotype C.

### 2.4. Construction of Chimeric Virus

Chimeric viruses containing the predominant ORF2–6 haplotypes were generated in the backbone of the infectious molecular clone pFL12 [51] using shuttle plasmids to facilitate swapping regions of pFL12 and the ORF2–6 haplotypes. The ORF2–6 sequences were selected from haplotypes existing in our library or synthesized (GeneArt, Thermo Fisher Scientific), inserted into the pFL12 backbone and transformed in DH5α *E. coli* cells. Chimeric viruses (designated with a “v” prior to the haplotype name, e.g., v3197A) were generated from the chimeric infectious clones via in vitro transcription and electroporation into MARC-145 cells, as described in [51]. Briefly, plasmid DNA was linearized by digestion with AclI and viral RNA was synthesized using the T7 Ultra mMESSAGE mMACHINE in vitro transcription kit (Ambion, Life Technologies, Carlsbad, CA, USA). Five μg of in vitro transcripts and 5 μg naïve MARC-145 cellular RNA were added to 2 × 10^6^ MARC-145 cells in 400 μL DMEM containing 1.25% DMSO and electroporated at 250 V and 950 uF (GenePulser Xcell, Bio-Rad, Hercules, CA, USA). Electroporated cells were plated in a single well of a 6-well plate in 5 mL DMEM supplemented with 10% fetal bovine serum (FBS), antibiotics, and 1.25% dimethyl sulfoxide (DMSO). At 18 h post transfection (hpt), media was replaced with 5 mL DMEM supplemented with 5% FBS and antibiotics. At 96 hpt, supernatants were harvested and cells were stained by immunocytochemistry to verify virus replication. Supernatants were passaged two to three times in MARC-145 cells to produce high titer chimeric virus stocks. All stocks were sequenced through ORF2–6 to confirm the correct haplotype sequence.

### 2.5. Virus Neutralization Assays

Neutralizing antibody assays were performed using a focus-reduction assay adapted from Wu et al. [54]. Briefly, sera was heat-inactivated, diluted, incubated for 1 h at 37 °C with 200 focus-forming units (FFU) of virus, and inoculated in duplicate or triplicate onto MARC-145 cells seeded the previous day in a 12-well plate at 3 × 10^5^ cells/well. Cells and virus were incubated an additional 24 h at 37 °C in 5% CO_2_, then the cells were fixed in ice-cold acetone:methanol and stained for PRRSV N protein by immunocytochemistry using the monoclonal antibody SDOW17 (RTI, LLC, Brookings, SD, USA) as the primary antibody and sheep anti-mouse IgG conjugated horse radish peroxidase (HRP) (Jackson ImmunoResearch, West Grove, PA, USA) as the secondary antibody. Following addition of the HRP substrate, cells were rinsed with distilled water, air-dried, and foci of infected cells enumerated by light microscopy. The percent reduction in FFU compared to a virus-only control was calculated as the percent neutralization. Assays were done in duplicate and repeated at least twice. 

Autologous sera samples from PHGC pigs were kindly provided by Drs. J.K. Lunney and R.R.R. Rowland and Type II PRRSV broadly neutralizing antiserum was a gift from Harrisvaccines, Ames, IA, USA.

### 2.6. PRRSV Binding and Entry Assays

To assess binding/attachment of PRRSV to MARC-145 cells, vFL12 was incubated in the presence or absence of neutralizing antibody for 1 h at 37 °C. The antibody source was a 1:2 dilution of pooled sera collected at 42 dpi from ~200 pigs experimentally infected with NVSL97-7895. The pooled sera sample was found to neutralize 86% of vFL12 at 1:8 dilution. The samples were chilled on ice and inoculated onto MARC-145 cells at a multiplicity of infection (MOI) of 1, and cells were incubated at 4 °C for one hour to facilitate virion attachment, but not uptake into the cells. Following incubation, media was removed, cells were washed six times with media and bound virus was eluted by incubating cells in 300 μL trypsin-ethylenediaminetetraacetic acid (EDTA) (1X solution, Sigma Scientific) for 10 min at room temperature. Following the addition of 50 μL FBS, cells and supernatant were collected and separated by centrifugation. Virion RNA was isolated from the supernatant fraction using the QIAamp Viral RNA Mini kit (Qiagen) and RNA was quantified by reverse transcriptase quantitative PCR (RT-qPCR) using primers specific for PRRSV ORF7 RNA.

For entry and replication assays, PRRSV was incubated in the presence or absence of antisera and inoculated onto MARC-145 cells at 4 °C as described above. Following washing to remove unattached virions, fresh media was added and the cells were shifted to 37 °C, designated as time 0 h. At 1, 4, 8, 12, or 24 h, cells were treated with trypsin and pelleted by centrifugation as described above, and total RNA was isolated from the cell fraction with the RNeasy mini kit (Qiagen) and quantified by RT-qPCR as above. All assays were done in duplicate and repeated 2–3 times and results are reported as mean copy number of viral RNA/well. 

### 2.7. Virus Replication Assays

Stocks of vFL12 and chimeric viruses were generated from p2 stocks by inoculation onto MARC-145 cells at an MOI of 0.001. Cells and virus were incubated 1 h at 37 °C at which time the inoculum was aspirated, cells washed two times and fresh media was added. Cells were washed again at 24 h post-infection, and p3 supernatant collected at 72 h post-infection was aliquoted and assayed for virus titer and virion copy number. Ten-fold serial dilutions of p3 stocks were inoculated onto MARC-145 cells that had been seeded the previous day at 3 × 10^5^ cells per well in 12-well plates. Following incubation at 37 °C for 24 h, cells were fixed in 50% acetone:50% methanol and foci of PRRSV infected cells were detected by immunocytochemistry as described above. Each FFU corresponds to a single infectious unit, and virus titer was calculated as FFU/mL of p3 stock. To determine virion copy number, RNA was isolated from p3 stocks using the QIAamp Viral RNA Mini Kit (Qiagen) and viral RNA was quantified using the VetMAX NA and EU PRRSV ORF7-specific RT-qPCR kit (Applied Biosystems, Thermo Fisher Scientific). Infectivity of each p3 stock is reported as the particle:infectivity ratio, calculated by dividing the least square mean for virus copy number by its corresponding least square mean for virus titer.

### 2.8. Statistical Analyses

A student’s *t*-test was used to compare percent neutralization of vFL12 to each chimeric virus and to analyze viral copy number at each time point in the presence or absence of neutralizing antibody. Replication phenotypes were analyzed using SAS 9.4 (Statistical Analysis System Institute Inc., Cary, NC, USA). Virus copy number was analyzed using a mixed model with the fixed class effects of virus, dilution (3 levels: 1:10, 1:50, or 1:100), and their interaction. Experiment (four levels) was fitted as a random effect to account for experimental variation. Virus titer was analyzed using a mixed model with the fixed class effects of virus and technician (two levels) and experiment (eight levels) fitted as a random effect. For each virus, infectivity was calculated as the ratio of virus copy number to viral titer using least square means obtained from analysis of each trait separately. The standard error of infectivity was calculated as the standard deviation of copy number to viral titer divided by the square root of the number of samples collected on each virus. Pairwise comparisons between all viruses, and comparisons of vFL12 with each chimeric virus, were then assessed using a student’s *t*-test. 

For all analyses presented, a threshold of *p* = 0.05 was used to determine statistical significance. 

### 2.9. Nucleotide Sequence Accession Numbers

The GenBank Accession numbers for the nucleotide sequences are KX286534-KX286735.

## 3. Results

### 3.1. Limited Genetic Variation at Early Times after Experimental PRRSV Infection

Sera samples from five pigs with detectable viremia at 5–6 weeks following experimental infection with NVSL97-7895 were used to analyze genetic variation in ORF2–6 at early times after infection. Viral RNA was isolated from the NVSL97-7895 inoculum and from sera samples collected at 7 dpi and at a late viremic day from each pig. Approximately 3 kb of ORF2–6 were amplified, TA-cloned, and up to 31 clones from each sample were sequenced. Multiple sequence alignments of all nucleotide sequences were generated by ClustalW to determine the amount of ORF2–6 variation in vivo (Table 1). In all pigs, the average pairwise identity within each sample was greater than 99%, similar to that observed in the starting inoculum. This high level of genetic identity revealed that very little genetic variation occurred in vivo during the six weeks following experimental PRRSV infection.

### 3.2. Location and Patterns of Genetic Variation in ORF2–6 at Early Times after Infection

**Figure 2:**
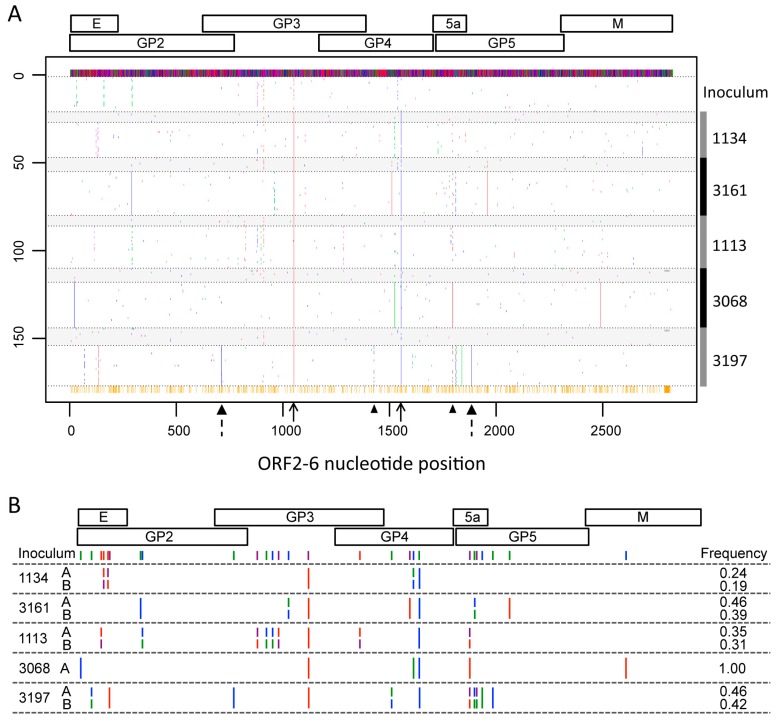
Variation in PRRSV open reading frame (ORF)2–6 following experimental infection. (**A**) ORF2–6 sequences are aligned to the consensus sequence of the inoculum (top line), with sites of variation from the inoculum consensus indicated by a colored dots. Sequences of individual clones are arranged vertically by inoculum and by pig (legend on the right). The dpi within a pig is separated by dotted horizontal lines, with seven dpi sequences shaded in gray. Numbers on the left denote the number of sequences aligned. Colors in the plot indicate nucleotide: A = green, G = blue, C = purple, T = red, and deletion = gray. Orange ticks at the bottom of the alignment indicate sites with at least one mutation across all clone sequences from all pigs, including possible deletions. Solid arrows denote sites of variation common across the majority of dpi and pigs. Dashed arrows indicate sites of variation within all clones in the late day sample from pig 3197. Arrowheads indicate linked sites of variation in late day sample from pig 3197. (**B**) Schematic of predominant ORF2–6 haplotypes constructed from the late day virus within each pig. Small colored ticks indicate sites where haplotypes differ from each other. Longer ticks indicate sites where haplotypes differ from the inoculum, but not from each other. Nucleotide colors are consistent with (A). The frequency of each haplotype is shown.

Due to the sequential sampling times within pigs, we were able to observe changes in the frequency of single nucleotide variants (SNVs) across sampling days. To visualize variation, the ORF2–6 sequences from the NVSL97-7895 inoculum and each pig sample were aligned relative to the consensus sequence of the inoculum (Figure 2A). Variation was observed in all open reading frames and, with the exception of two changes that were observed in all pigs (Figure 2A, solid arrows), the sites of variation differed across pigs. Within each individual pig, we identified variable sites with dominant changes present in all clones in a given sample (Figure 2A, dashed arrows). Interestingly, several sites of variation within a given sample appeared to be linked (Figure 2A, arrowheads), raising the possibility that effects of variation on viral phenotype may be influenced by epistatic interactions between/among different viral envelope proteins. In addition to changes at highly variable sites, there were numerous SNVs that were observed only in single clones, likely reflecting random variation. The most striking observations from these analyses was that virus variation was largely pig-specific and no single variant, or pattern of variation, clearly distinguished virus isolated from pigs with rebound viremia (1113, 3068, 3197) and virus from pigs with prolonged viremia (1134, 3161). 

### 3.3. Identification of Pig-Specific ORF2–6 Haplotypes within Late Day Virus Populations

The major and minor PRRSV envelope proteins interact to form oligomeric complexes on the virion surface, and it is possible that variation at a particular site in one envelope protein may affect or constrain variation at a second site in the same or different envelope protein. Because we utilized single clone sequencing, where each clone represents a single viral genome, it was possible to identify linked sites of variation across ORF2–6 (Figure 2A). Based on these linked sites, we identified unique viral haplotypes, where a haplotype refers to the set of SNVs on an individual genome (Figure 2B). In four of the five pigs, two predominant haplotypes co-existed at relatively similar frequencies (Figure 2B), while a single haplotype representing the consensus sequence was found in pig 3068. In addition to the predominant haplotypes shown in Figure 2B, all samples contained minor haplotypes that were present at lower frequencies, and that often comprised combinations of the predominant haplotypes (not shown). Additional SNVs and haplotypes are present in the population, but were not detected using our sequencing strategy.

**Figure 3:**
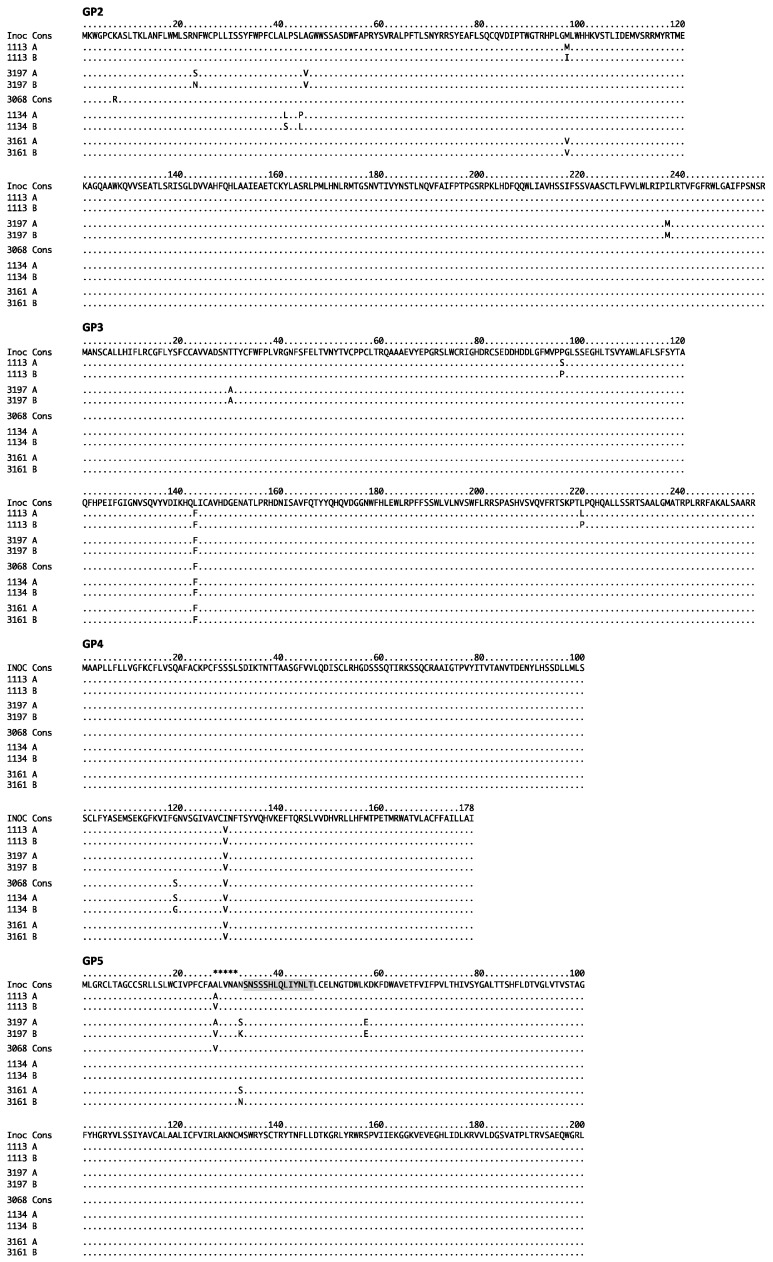
Amino acid sequence variation in the major and minor glycoproteins encoded by ORF2–6. The amino acid sequences of predominant haplotypes in the five experimentally-infected pigs are aligned to the consensus sequence of the inoculum. The amino acid is shown for sites where the haplotype differs from the inoculum and/or from each other. Dots (.) indicate an identical amino acid to the reference sequence in both haplotypes. Shaded area denotes location of reported neutralizing epitope in GP5 [16,18]. Asterisks in GP5 indicate location of the decoy epitope [36]. Numbering at the top of the alignment is based on translational start site of each glycoprotein.

The non-synonymous SNVs and associated amino acid sequences for the predominant ORF2–6 haplotypes are shown in Tables S1 and S2 and in Figure 3. While some ORF2–6 SNVs were shared across virus samples in different pigs, the predominant haplotypes were pig-specific. Some haplotypes included SNVs that were detected in the inoculum and/or seven dpi samples, while other SNVs were present in only the late dpi sample (Tables S1 and S2). It is not known if the SNVs appearing only in late dpi samples arose de novo during the course of infection, or were present at low frequency in the inoculum. Importantly, none of the predominant haplotypes detected in late day viremic periods were observed in any of the inoculum clones. The fact that late day virus populations were characterized by the predominance of distinct haplotypes suggests that, although there was limited variation overall, the changes that did occur may be biologically important for virus replication at early times after infection. 

### 3.4. Variation in ORF2–6 Increases Resistance to Neutralizing Antibody in Some, but Not All, Pigs

**Figure 4:**
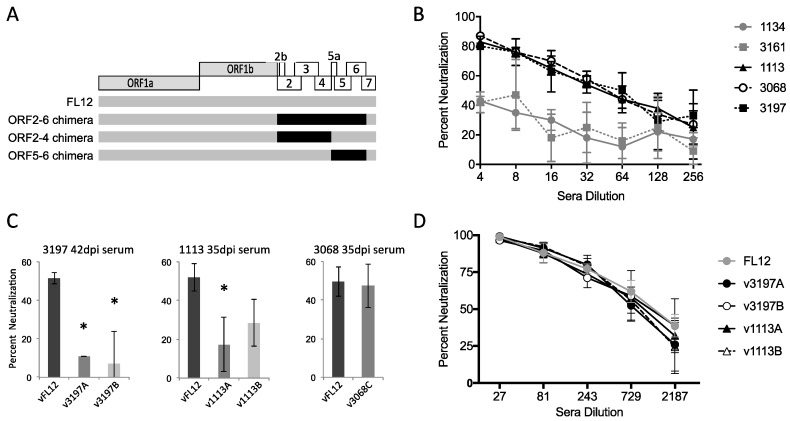
Neutralization phenotype of ORF2–6 haplotypes: (**A**) Schematic of chimeric viruses containing rebound haplotypes. (**B**) Virus neutralizing activity of 42 dpi pig sera from each of the five pigs against the inoculum virus. (**C**) Susceptibility of vFL12 and ORF2–6 chimeric viruses to neutralization by autologous sera diluted 1:4 (1113 and 3068) or 1:8 (3197). Asterisks indicate significant difference (*p* < 0.05) in neutralization compared to vFL12. (**D**) Susceptibility of vFL12 and ORF2–6 haplotype to neutralization by broadly neutralizing PRRSV anti-sera. Results denote the mean percent neutralization ± SD compared to a no-serum control.

To investigate the possibility that limited genetic variation may nonetheless alter important virus phenotypes, the predominant ORF2–6 haplotypes from each of the five experimentally infected pigs were used to generate ORF2–6 chimeric viruses in the background of vFL12, which was derived from the NVSL97-7895 inoculum (Figure 4A, Table S3). Chimeric viruses were tested for effects of variation on antigenic and replication phenotypes that might contribute to virus replication in vivo. To ascertain if genetic variation altered susceptibility to virus neutralizing antibody, we first determined if any of the five experimentally infected pigs had developed detectable neutralizing antibody to the inoculum virus, NVSL97-7895, by 42 dpi (Figure 4B). Although no sera was able to neutralize 100% of the inoculum virus, three pigs (1113, 3068, and 3197) were able to neutralize over 75% of inoculum virus at a 1:8 serum dilution. In contrast, pigs 1134 and 3161 had no detectable neutralizing activity against the inoculum virus, even though they were both cleared of virus by 42 dpi. Of note, the pigs with detectable neutralizing activity were those three that experienced a rebound in viremia, raising the possibility that ORF2–6 haplotypes associated with rebound viremia could be immune escape variants. To explore this, chimeric viruses containing predominant ORF2–6 haplotypes from the three pigs with detectable neutralizing antibody to the inoculum virus were tested for sensitivity to neutralization by autologous serum collected the same day that rebound viremia was detected (Figure 4C). The pFL12 infectious molecular clone represents the consensus sequence of the inoculum virus, NVSL97-7895 [51], and neutralization of vFL12 was used as a reference in all neutralization assays. Due to the limited amount of pig sera available, all assays were done using a 1:4 or 1:8 dilution of autologous sera, which neutralized ~50% of vFL12. 

In two of the three pigs with neutralizing antibody to the inoculum virus, predominant ORF2–6 haplotypes from rebound viremia were found to confer increased resistance to neutralization by autologous sera (Figure 4C). Chimeric viruses containing either haplotype A or haplotype B from pig 3197, designated v3197A or v3197B, were significantly more resistant to neutralization by autologous sera than was vFL12, with average neutralization of 11% (*p* = 0.0002) and 7% (*p* ≤ 0.001), respectively. Chimeric virus containing haplotype A from pig 1113, v1113A, was also significantly more resistant to neutralization than vFL12 (average neutralizations of 18%, *p* = 0.009). Chimeric virus containing haplotype B from pig 1113, v1113B, showed increased resistance to neutralization, however this difference was only close to being significant (average neutralization of 29%, *p* = 0.059). In contrast, chimeric virus containing the single dominant ORF2–6 haplotype from pig 3068 (v3068C) was neutralized at levels similar to vFL12 (48%, *p* = 0.8). Each of the four chimeric viruses with increased resistance to autologous sera was also tested for sensitivity to neutralization using sera with broadly neutralizing activity against Type II PRRSV (Figure 4D). All were highly susceptible to broadly neutralizing sera, as was vFL12. Together, these results indicate that detectable neutralizing antibody is not required for control of viremia in PRRSV-infected pigs. However, in some, but not all pigs with neutralizing antibody to the inoculum virus, genetic changes in ORF2–6 can alter sensitivity to autologous, type-specific neutralizing antibody.

### 3.5. Variation in Either Major or Minor Envelope Glycoproteins Can Mediate Escape from Autologous Neutralizing Antibody

**Figure 5:**
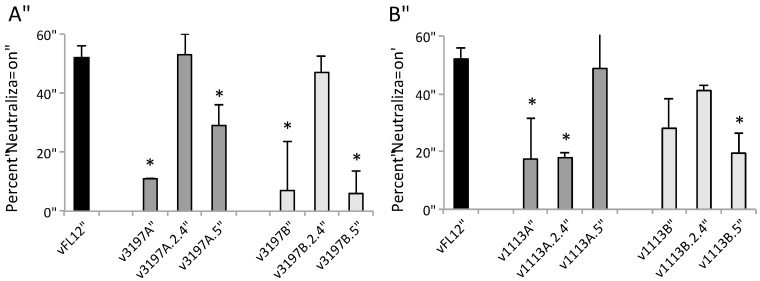
Mapping regions of ORF2–6 that confer resistance to autologous neutralizing antibody in (**A**) pig 3197 and (**B**) pig 1113. Two hundred focus-forming units (FFU) of vFL12 or chimeric viruses containing either ORF2–4 of ORF5 haplotypes were tested in neutralization assays using autologous sera. Results denote the mean percent neutralization ± standard deviation (SD) compared to a no-serum control. Asterisks (*) indicate significant reduction (*p* < 0.05) in percent neutralization as compared to vFL12.

To identify the envelope protein(s) that contributed to increased resistance to neutralization by autologous sera, we generated a set of chimeric viruses in which the predominant haplotypes from pigs 3197 and 1113 were separated into their oligomeric units (Figure 4A, Table S3). None of these haplotypes contained amino acid changes in M (Table S1), so the chimeric virus designations include either 2–4 or 5 (Figure 5). As before, each of the chimeric viruses was tested for sensitivity to neutralization by autologous sera, using vFL12 as a reference in all neutralization assays. In pig 3197, both v3197A-5 and v3197B-5 viruses were significantly more resistant to neutralization by autologous sera than vFL12, with average neutralizations of 29 and 6% (*p* = 0.035 and <0.001), respectively (Figure 5A). In contrast, chimeric viruses containing the ORF2–4 region (v3197A-2-4 and v3197B-2-4) were neutralized at levels similar to vFL12. Thus, increased resistance to neutralization in both v3197A and v3197B mapped to ORF5. There are three sites in GP5 at which the 3197A and/or 3197B haplotypes differ from vFL12: amino acid positions 27, 32, and 57 (Figure 3). An alanine is found at position 27 in vFL12 and in the 3197A haplotype, while 3197B contains a valine at this position. A K57E change is found in both 3197A and 3197B, and each haplotype differs from vFL12 at GP5 residue 32: 3197A contains an N32S mutation, while 3197B contains an N32K mutation. The N32K mutation is coincident with a Q36K change in protein 5a, which overlaps GP5 (Table S1). Protein 5a is a very minor component of the virion and previous studies found that immunization with 5a does not elicit neutralizing antibodies [34,35]. 

In pig 1113, haplotypes A and B differed in respect to the region that mediated increased resistance to neutralization (Figure 5B). Chimeric virus v1113A-2-4 was neutralized at 18%, (*p* = 0.016) whereas v1113A-5 was neutralized at levels similar to vFL12, indicating that resistance to neutralization in the 1113A haplotype mapped to ORF2–4. In contrast, increased resistance of v1113B was mediated through ORF5: v1113B2-4 chimeric virus was neutralized at similar levels as vFL12 (41%, *p* = 0.2), but v1113-B-5 chimeric virus was significantly more resistant to neutralization (19%, *p* = 0.025) (Figure 5B). There are three sites in ORF2–4 where the 1113A haplotype differs from vFL12: P96S and L143F in GP3 and I129V in GP4. Two of these changes, GP3 L143F and GP4 I129V, were found in all pigs, whereas the GP3 P96S change was unique to the 1113A haplotype. The only amino acid difference between v1113B-5 and vFL12 is the A27V substitution in GP5 (Figure 3), indicating that a single point mutation can increase resistance to autologous neutralizing antibody. 

Because of the limited variation in ORF2–6, it was of interest to determine if other single amino acid changes conferred resistance to autologous neutralizing antibody. Viruses were generated that contained unique amino acid changes found only in neutralization resistant haplotypes: GP3 P96S, GP5 N32K, or GP5 K57E. Individually, none of these single amino acid changes resulted in increased resistance to autologous neutralizing antibody (not shown). The GP5 A27V substitution was found in predominant genotypes from all three pigs that had neutralizing antibody to the inoculum virus (1113, 3197, 3068) (Figure 3). As noted above, the GP5 A27V substitution alone was sufficient to confer resistance to neutralization by pig 1113 autologous sera (Figure 5B); however, this was not true for 3197 autologous sera (data not shown). In addition, the presence of GP5 A27V in the 3068C haplotype did not confer resistance to 3068 neutralizing sera (Figure 4C). In most cases, therefore, resistance or sensitivity to neutralization depended on a combination of amino acid changes that were unique to each haplotype, and to each pig. Importantly, there was no single amino acid change that was predictive of neutralization phenotype.

Overall, results of these mapping studies revealed that variation in ORF2–4 or ORF5 could, independently, confer increased resistance to neutralization. Because the minor and major glycoproteins are believed to play different and/or distinct roles during early stages of virus replication [15,45,55], these findings raise the possibility that PRRSV is susceptible to neutralization at multiple steps in the virus replication cycle. 

### 3.6. The Effects of Neutralizing Antibody at Early Steps in the PRRSV Replication Cycle

**Figure 6:**
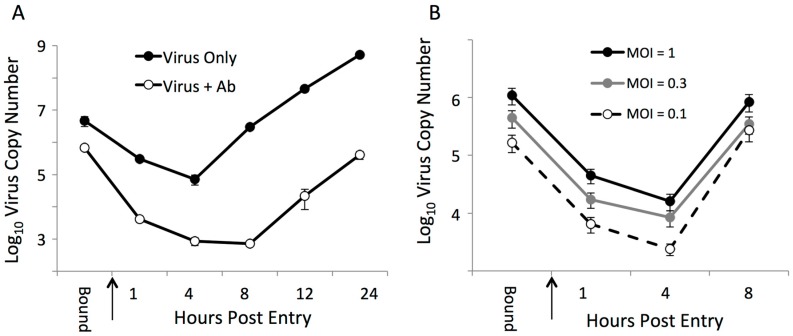
The effect of neutralizing antibody at early stages of PRRSV replication. Quantitation of PRRSV vFL12 binding and entry: (**A**) in the presence and absence of neutralizing antibody; and (**B**) in cells inoculated at varying multiplicity of infection (MOI). Bound represents the RNA copy number present in attached virions after incubation at 4 °C, and the arrow indicates time 0, when cells were shifted to 37 °C. Results denote the mean virus copy number per well ± standard error of the mean (SEM).

Mapping studies indicated that targets of virus-neutralizing antibody include both the major and minor glycoprotein complexes. To better understand how variation in PRRSV glycoproteins contributes to increased resistance to neutralization, we quantified the effects of neutralizing antibody at early steps in PRRSV replication (Figure 6). For these assays, we used pooled sera collected at 42 dpi from ~200 pigs experimentally infected with NVSL 97-7895. This sera is expected to have broad specificity, and was found to neutralize all chimeric viruses at similar titers (data not shown). Binding/attachment of PRRSV to MARC-145 cells was significantly reduced in the presence of virus-neutralizing antibody (*p* < 0.05) (Figure 6A). Following entry, detectable virus RNA decreased in both treatment groups from 1–4 h post-entry, indicative of the eclipse phase of virus replication. In the absence of antibody, production of new virion RNA occurred between 4 and 8 h post-entry and rapidly increased through 24 h post-entry. In the presence of neutralizing antibody, however, newly synthesized viral RNA was not detected until after 8 h post-entry, after which time the rate of increase in viral RNA was similar to that seen in the absence of antibody. The delayed appearance of newly synthesized RNA was not merely a consequence of reduced virion attachment, as the cells that were infected at different MOIs showed similar kinetics during the eclipse phase, and synthesis of viral RNA was always initiated between 4 and 8 h post-entry (Figure 6B). In addition to blocking attachment, therefore, neutralizing antibody also targets a post-entry step in virus replication that occurs between 4 and 8 h post-entry. It is not clear from these data which of the viral proteins are targeted at the attachment and/or post-entry steps in virus replication. Nonetheless, these data provide support for the mapping results indicating that virus-neutralizing antibody may target both the major and minor glycoproteins to inhibit multiple steps during early stages of PRRSV infection.

### 3.7. The Effect of ORF2–6 Variation on PRRSV Replication Phenotype

**Figure 7:**
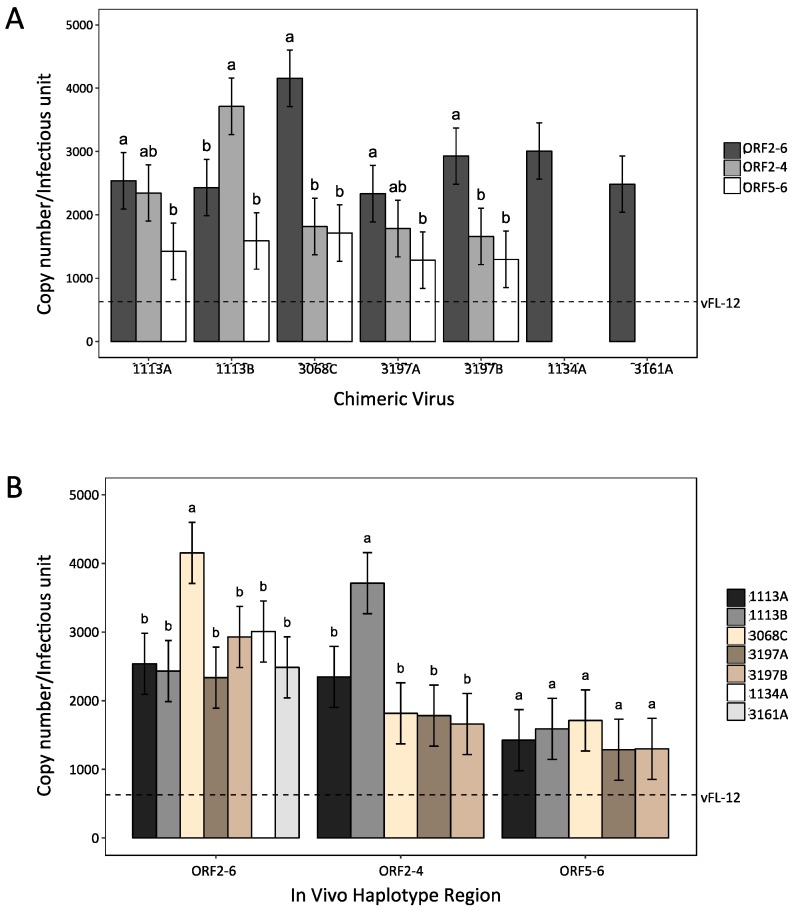
Infectivity of ORF2–6 chimeric viruses on MARC-145 cells: (**A**) The effect of ORF2–6 regions from different haplotypes on infectivity of MARC-145 cells. Within a haplotype, different letter assignments significantly differ at *p* < 0.05. (**B**) Comparison of infectivity on MARC-145 cells among chimeric viruses containing different ORF2–6 haplotypes, or regions within the ORF2–6 haplotypes. Within each group (ORF2–6, ORF2-4, and ORF5-6), viruses with different letter assignments significantly differ at *p* < 0.05. Dotted line indicates the infectivity of vFL12.

The result of our immunological analyses indicated that variation in ORF2–6 can result in antigenic variation and increased resistance to neutralization by autologous sera. However, this was not the case in three of the five pigs, two of which (1134 and 3161) had no detectable neutralizing antibody to the inoculum virus. Variation in ORF2–6 has been shown to occur during sequential in vitro passage and attenuation of PRRSV [49,50], and it is possible that some of the observed variation in ORF2–6 altered virus replication phenotype. To explore this, stocks of chimeric viruses were assayed for virus titer and virion copy number (Figure S1) and the particle: infectivity ratio was calculated for each of the chimeric viruses (Figure 7). Genetic variation in ORF2–6 resulted in significant differences in infectivity on MARC-145 cells (Figure S1). Most notably, vFL12 was significantly more infectious for MARC-145 cells than all of the chimeric viruses that contained ORF2–6 (*p* < 0.0005), ORF2–4 (*p* < 0.05) and two of the viruses that contained ORF5–6 (1113B-5 and 3068C-5, *p* < 0.05) haplotypes from experimentally infected pigs. Within each haplotype, the least infectious viruses were usually those that contained the complete ORF2–6 haplotype (Figure 7A). It is likely, therefore, that ORF2–6 variation early after experimental infection includes changes that increased adaptation to replication in vivo at a cost for replication in cell lines such as MARC-145. In support of this, the two amino acid changes shared by all predominant haplotypes, GP3 L143F and GP4 I129V (Figure 3), occurred at sites previously reported to vary during serial in vitro passage and/or attenuation of PRRSV field strains [49].

Few significant differences in infectivity were observed across the different haplotypes (Figure 7B). Haplotypes that were predominant in pigs with rebound viremia (1113, 3068, 3197) had similar infectivity as haplotypes from pigs with prolonged viremia (1134, 3161). The A and B haplotypes that were predominant in pigs 1113 and 3197 showed no differences in infectivity, and differences in infectivity among chimeric viruses were not associated with differences in the neutralization phenotype of the virus.

## 4. Discussion

Genetic diversity of porcine reproductive and respiratory syndrome virus (PRRSV) confounds efforts to develop effective and broadly acting vaccines. Genetic variation may lead to changes in virus phenotypes that are important in replication, immune evasion, host cell tropism and/or transmissibility; however, few studies directly link PRRSV genotype and phenotype. Here, sera samples from five experimentally infected pigs collected at early times after infection were used to characterize ORF2–6 variation and determine the effects of variation on the antigenic and replication phenotype of PRRSV. Limited genetic variation was observed during the first six weeks after infection. However, predominant ORF2–6 haplotypes were identified in each pig which, using reverse genetics, were found to vary in antigenic and/or replication phenotype. In some but not all pigs, genetic changes in ORF2–4 and/or ORF5 resulted in increased resistance to autologous, type-specific neutralizing antibody. Resistance or sensitivity to neutralization depended on a combination of amino acid changes that were unique to each pig, and there was no single amino acid change that was predictive of neutralization phenotype. Rather, results suggest that virus-neutralizing antibody may target both the major and minor glycoproteins to inhibit multiple steps at early stages of PRRSV replication. In all five pigs, genetic variation in ORF2–6 resulted in significant reduction in infectivity on MARC-145 cells, suggesting variation in ORF2–6 may also be important for virus replication in vivo. Together, these data reveal that variation appearing early after infection alters important virus phenotypes and contributes to antigenic and biologic diversity of PRRSV. 

In two of the five pigs, chimeric viruses that contained ORF2–6 haplotypes that were predominant during a period of rebound viremia conferred increased resistance to neutralization by autologous sera that neutralized the inoculum virus. These findings provide experimental evidence that immune pressure by neutralizing antibody can alter the neutralization phenotype of PRRSV in vivo. Immune escape from neutralizing antibody has been previously associated with genetic changes in GP4 and GP5 of Type I and Type II PRRSV, respectively [9,56,57]. Costers et al. [9,56] identified amino acid substitutions in the GP4 neutralizing epitope of Type I strains that arose during immune selection in vitro or in vivo, and resulted in immune escape. Type II strains do not contain an analogous GP4 neutralizing epitope, but a neutralizing epitope has been identified in the GP5 ectodomain of Type II stains [16,18]. However, strong evidence that amino acid substitutions in this epitope give rise to immune escape variants in vivo is lacking [58]. Amino acid substitutions at positions 102 and 105 in the C-terminal region of GP5 occur following immune selection in vivo [21,57], and it was suggested that the GP5 neutralizing epitope may be conformational, rather than linear [23,57]. In the present study, changes within neutralization epitopes did not always lead to increased resistance to neutralization, and occurred in pigs that lacked detectable neutralizing antibody (i.e., pig 3161). In the three pigs with neutralizing antibody, amino acid substitutions were found at A27 and/or N32 in GP5. These sites are located in the GP5 ectodomain, and variation at these positions can alter GP5 peptide signal processing or *N*-linked glycosylation [6,7,16,59]. In addition, a recent study identified these positions as sites of diversifying selection early after PRRSV infection [60], suggesting these sites may be targets of immune selection. However, amino acid substitutions A27 and/or N32 in GP5 were present in both neutralization-resistant and neutralization-sensitive ORF2–6 haplotypes (Figure 3), indicating that variation at these sites was not predictive of neutralization phenotype. A single A27V substitution in GP5 led to increased neutralization resistance in the v1113B haplotype, but this was not the case with other ORF2–6 haplotypes. Indeed, with the exception of GP5 A27V in 1113B, no single amino acid change analyzed was sufficient to confer increased neutralization resistance. Therefore, sensitivity or resistance to neutralization appeared to depend on the repertoire of amino acid substitutions, rather than the presence of a single amino acid substitution. Importantly, the repertoire and pattern of amino acid substitutions in ORF2–6 was different in each of the five pigs and was independent of the presence or absence of neutralizing antibody. A consequence of the unique, pig-specific patterns of neutralization resistance is a lack of robustness. All ORF2–6 haplotypes that increased resistance to concomitant autologous sera were easily neutralized by broadly neutralizing sera (Figure 4D), pooled sera, or by autologous sera collected later in infection (not shown). The presence of high-titered, cross-reacting neutralizing antibody in commercial sows [61] suggests that, over time, exposure to increasingly diverse virus populations in vivo can result in a stronger, broader, and more effective host immune response to PRRSV.

Models of the early steps of PPRSV replication were originally developed by analysis of virus replication in macrophages in vitro [15]. In these models, the initial steps of attachment and internalization are mediated by interactions between the GP5/M heterodimer and attachment factors on the cell surface, including heparan sulfate and CD169. The initial interactions are thought to enhance binding of the GP 2/3/4 trimer to the primary PRRSV receptor, CD163, which is located on both the cell surface and in the endosome. Based on these models, neutralizing antibody targeted to GP5 would be predicted to inhibit attachment and entry, whereas antibody targeted to GP2/3/4 would inhibit later steps in replication, including fusion and/or uncoating. In our studies, comparison of PRRSV replication in the presence and absence of neutralizing antibody revealed a modest but significant block in attachment. In addition, there was a delay in the appearance of newly synthesized viral RNA, with significantly reduced levels of virus RNA through 24 h post-entry. The delay in synthesis of viral RNA was not due to lower levels of attachment, indicating that neutralizing antibody can also inhibit a second, post-entry step in PRRSV replication. The antisera used in these assays was pooled sera collected from ~200 pigs at 42 days after infection with NVSL97-7895, the same inoculum used in the five pigs analyzed in this study. It is expected that the targets of neutralizing antibody in this pool would be similar to what we observed in pigs 1113 and 3197, and include both the major and minor envelope glycoproteins. Based on models of PRRSV entry cited above, it is tempting to speculate that antibody targeted to GP5 inhibited attachment, while the post-entry inhibition was due to antibody targeted to GP2/3/4. A caveat to this interpretation is that MARC-145 cells do not express CD169, and CD169 was recently shown to be dispensable for PRRSV replication in vivo [42]. It is possible that antibody targeted to GP5 blocks interaction with another cellular protein important in virus replication. For example, a recent study reported that MYH9 interacts with GP5 and is an essential factor in PRRSV replication [47]. Due to the limited amount of available sera, we are unable to explore these possibilities using our panel of chimeric viruses and autologous sera. The finding that PRRSV-infected pigs generate a neutralizing antibody response that targets both GP5 and GP2/3/4, and one that inhibits multiple stages in early virus replication, may motivate additional studies to delineate mechanism(s) by which neutralizing antibody inhibits PRRSV replication.

In vivo fitness of PRRSV within an individual host, or herd, depends on a number of interacting factors, including replication fitness, immune evasion, cell tropism, and transmissibility. Experimental analyses of replication fitness can be measured in vivo, but most studies rely on in vitro surrogates, such as replicative capacity and/or infectivity in cell culture. An added complication is the limited cell tropism of PRRSV. Replication of PRRSV in vitro is limited to differentiated macrophages such as porcine alveolar macrophages (PAM) or MARC-145 and other MA104-derived monkey kidney cell lines. Primary PAM cultures can be established in vitro, but the cultures are short lived and there is great heterogeneity within and between different PAMs that confounds reproducibility of experimental results. These difficulties have contributed to our limited knowledge regarding the link between PRRSV genotype and replication phenotype. Some insight has been gained by characterizing viral populations during sequential passage of field isolates in vitro. Sequence analysis of viral population at different passage levels, as well as comparisons among different attenuated vaccine viruses with their parental strains, has revealed common sites of amino acid variation that arose during in vitro selection [49,50]. Based on these analyses, it is likely that at least some of the ORF2–6 variation observed in this study reflects selection for replication in vivo. Two amino acid substitutions in ORF2–6 were observed in all five of the experimentally infected pigs: GP3 L143F and GP4 I129V. In both cases, the change resulted in reversion to an amino acid found in field strains PRRSV [49]. Moreover, chimeric viruses containing ORF2–6 haplotypes from each of the five pigs were found to be significantly less infectious for MARC-145 cells than vFL12, and mapping studies revealed that changes in either or both major and minor glycoproteins contributed to replication phenotype in MARC-145 cells. It is not clear how the specific substitutions in GP3 and GP4 affect PRRSV replication phenotype, or what other changes observed in ORF2–6 might contribute to virus replication in vivo. It is possible that one selective factor is cell tropism [62]. As noted above, MARC-145 cells lack the CD169 receptor found in PAM, and in vitro passage selects for viruses able to replicate in the absence of CD169. Although CD169 is not required for PRRSV replication in vivo, the presence of both receptors enhances in vitro replication of Type I PRRSV strains [45]. Some of the observed variation in ORF2–6 may reflect genetic adaptations to virus replication in the presence or absence of CD169. 

Genetic diversity is a hallmark of PRRSV infection in vivo, and it was not surprising to find SNVs in all proteins encoded by ORF2–6. For the most part, sites of variation differed across pigs; however, within individual pigs, we observed highly variable sites in all, or nearly all, sequenced clones. In four of the five pigs analyzed, predominant haplotypes co-existed, indicative of the quasispecies population structure of PRRSV in vivo. Within a quasispecies, interactions between or within variant genomes collectively contribute to the overall characteristics that impact virus evolution and pathogenesis. In an effort to capture potential interactions within/between PRRSV ORF2–6 genotypes, we chose to clone and sequence individual viral genomes, rather than use high-throughput sequencing platforms that yield shorter read lengths. An advantage of our approach was the ability to discern distinct viral haplotypes that co-existed within an individual pig. Moreover, using reverse genetics, we demonstrated that individual haplotypes could have different, and potentially complementary, phenotypes predicted to enhance virus replication in vivo. It is not practical to phenotype each individual genotype within a viral quasispecies, and we recognize the limitations of sampling and characterizing only the predominant genotypes. It is hoped that continued progress in defining links between PRRSV genotype and biologically significant phenotypes, together with rapid advances in high-throughput sequencing technologies and computational tools, will increase our understanding of genetic and phenotypic diversity in PRRSV and aid efforts to control this significant swine pathogen.

## Figures and Tables

**Table 1:** Summary of sequenced clones and ORF2–6 (open reading frame) variation.

Sample ID	Viremia Classification	Day Post Inoculation	Number Clones Sequenced ^a^	Pairwise Identity ^b^
Inoculum	N/A	0	25	99.71
1134	Prolonged	7	8	99.76
		28	21	99.63
3161	Prolonged	7	9	99.71
		28	28	99.72
1113	Rebound	7	9	99.71
		35	26	99.62
3068	Rebound	7	9	99.40
		35	31	99.78
3197	Rebound	7	10	99.49
		41	26	99.76
	Combined		202	99.57 ^c^

^a^ Number of individual clones sequenced from each sample. ^b^ Average nucleotide pairwise identity across all clones from a sample. ^c^ Average nucleotide pairwise identity across all clones sequenced from each gene region.

## Supplementary Materials

The following are available online at www.mdpi.com/1999-4915/9/5/113/s1. Figure S1: Replication phenotype of chimeric virus containing predominant ORF2–6 haplotypes. Table S1: Amino Acid changes in ORF2–6 haplotypes from late day samples from pigs with rebound viremia. Table S2: Amino acid changes in ORF2–6 haplotypes predominant in late day samples from pigs with prolonged viremia. Table S3: List of chimeric viruses assayed for neutralization and/or replication phenotype.
