# Supplementary material for: Antigenic and Biological Characterization of ORF2–6 Variants at Early Times Following PRRSV Infection
Source: Viruses. 2017 May 16;9(5):113. doi: 10.3390/v9050113 (PMC5454425; doi:10.3390/v9050113)
Supplement: Supplementary file 1 [file viruses-09-00113-s001.pdf]

Supplementary Materials: Antigenic and Biological Characterization of ORF2-6 Variants at Early Times Following PRRSV Infection

Alyssa B. Evans, Hyelee Loyd, Jenelle R. Dunkelberger, Sarah van Tol, Marcus J. Bolton, Karin S. Dorman, Jack C. M. Dekkers and Susan Carpenter

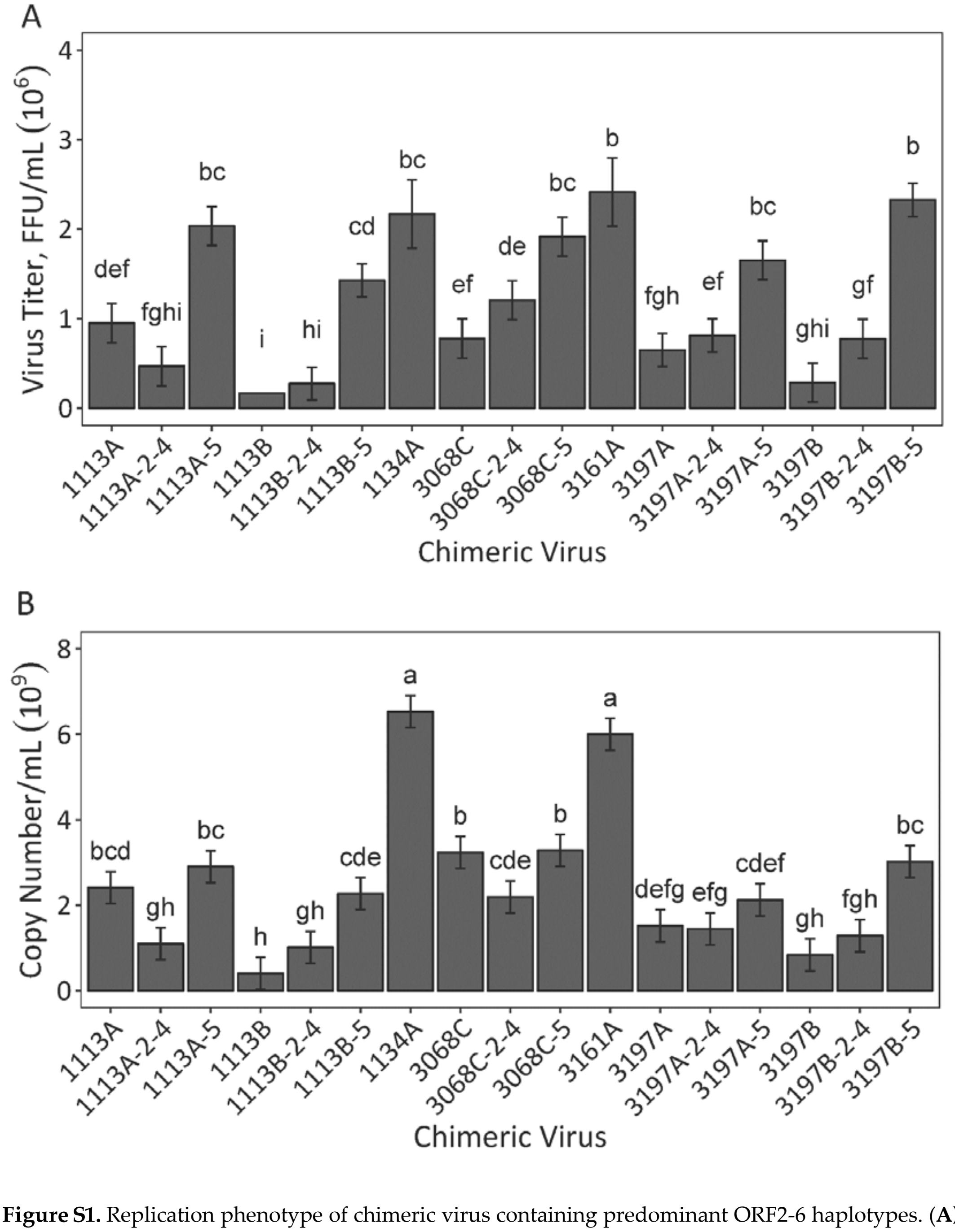

Figure S1. Replication phenotype of chimeric virus containing predominant ORF2-6 haplotypes. (**A**) Virus titers Stocks of chimeric viruses titered on MARC-145 cells and reported as focus-forming units per mL stock; (**B**) Viral RNA was purified from chimeric virus stocks and quantified by RT-qPCR (Applied Biosystems). Results are reported as the least square mean (± standard error) of virus titer and virion copy number. Pairwise comparisons between all viruses were assessed using a student's *t*-test and groups with different letter assignments significantly differ at $p < 0.05$.

Table S1. Amino Acid changes in ORF2-6 haplotypes from late day samples from pigs with rebound viremia.

	Position[a]			Haplotype A[b]		Haplotype B[b]		Inoculum[c]	
Pig	NT	AA		NT	AA	NT	AA	NT	AA
1113	114	GP2<br>E	38*<br>37	T	C<br>F	C	C<br>L	T	C<br>F
	291	GP2	97	G	M	A	I	G	M
	909	GP3	96	T	S	C	P	C	P
	1050	GP3	143	T	F	T	F	C	L
	1282	GP3<br>GP4	220<br>38*	T	L<br>T	C	P<br>T	T	L<br>T
	1553	GP4	129	G	V	G	V	A	I
	1795	GP5<br>5a	27<br>30*	C	A<br>C	T	V<br>C	C	A<br>C
3197	68	GP2	23	G	S	A	N	A	N
	134	GP2<br>E	45<br>43*	T	V<br>G	T	V<br>G	C	A<br>G
	711	GP2<br>GP3	237<br>30	G	M<br>A	G	M<br>A	A	I<br>T
	1050	GP3	143	T	F	T	F	C	L
	1553	GP4	129	G	V	G	V	A	I
	1795	GP5<br>5a	27<br>30*	C	A<br>C	T	V<br>C	C	A<br>C
	1810	GP5<br>5a	32<br>35*	G	S<br>Q	A	K<br>Q	A	N<br>Q
	1811	GP5<br>5a	32<br>36	C	S<br>Q	A	K<br>K	C	N<br>Q
	1838	GP5<br>5a	41*<br>45	A	L<br>N	A	L<br>N	G	L<br>D
	1884	GP5	57	G	E	G	E	A	K
3068	20	GP2<br>E	7<br>5*	G	R<br>Q	Not Applicable		A	K<br>Q
	1050	GP3	143	T	F			C	L
	1523	GP4	119	A	S			G	G
	1553	GP4	129	G	V			A	I
	1795	GP5<br>5a	27<br>30*	T	V<br>C			C	A<br>C
	2489	M	63	T	L			G	V

[a] The nucleotide (NT) position is based on the ORF2-6 region, with the first nucleotide of the GP2 start site designated 1. The amino acid (AA) denotes the associated protein, amino acid and amino acid position in the protein. Shaded regions indicate sites where a NT change occurs in an overlapping reading frame, and the amino acids changes and position is listed for reading frames. [b] Haplotypes A and B represent the haplotypes present at the highest and second highest frequencies within each pig, respectively. [c] The consensus nucleotide and amino acid in the inoculum. * Synonymous change.

Table S2. Amino acid changes in ORF2-6 haplotypes predominant in late day samples from pigs with prolonged viremia.

	Position[a]			Haplotype A[b]		Haplotype B[b]		Inoculum[c]	
Pig	NT	AA		NT	AA	NT	AA	NT	AA
1134	122	GP2<br>E	41<br>39*	T	L<br>F	C	S<br>F	T	L<br>F
	131	GP2<br>E	44<br>42*	C	P<br>A	T	L<br>A	T	L<br>A
	1050	GP3	143	T	F	T	F	C	L
	1523	GP4	119	A	S	G	G	G	G
	1553	GP4	129	G	V	G	V	A	I
3161	289	GP2	97	G	V	G	V	A	M
	1050	GP3	143	T	F	T	F	C	L
	1553	GP4	129	G	V	G	V	A	I
	1810	GP5<br>5a	32<br>35*	G	S<br>Q	A	N<br>Q	A	N<br>Q

[a] The nucleotide (NT) position is based on the ORF2-6 region, with the first nucleotide of the GP2 start site designated 1. The amino acid (AA) denotes the associated protein, amino acid and amino acid position in the protein. Shaded regions indicate sites where a NT change occurs in an overlapping reading frame, and the amino acids changes and position is listed for reading frames. [b] Haplotypes A and B represent the haplotypes present at the highest and second highest frequencies within each pig, respectively. [c] The consensus nucleotide and amino acid in the inoculum. * Synonymous change.

Table S3. List of Chimeric Viruses Assayed for Neutralization and/or Replication Phenotype.

Pig	Haplotype	ORF2-6[a]	ORF2-4[a]	ORF5-6[a]
1113	A	v1113A	v1113A-2-4	v1113A-5
	B	v1113B	v1113B-2-4	v1113B-5
1134	A	v1134A	ND[b]	ND[b]
3068	A	v3068C	v3068C2-4	v3068C-5
3161	A	v3161A	ND	ND
3197	A	v3197A	v3197A-2-4	v3197A-5
	B	v3197B	v3197B-2-4	v3197B-5

[a] Region of PRRSV genome contained in chimeric virus; [b] Not Done.

© 2017 by the authors. Submitted for possible open access publication under the terms and conditions of the Creative Commons Attribution (CC BY) license (http://creativecommons.org/licenses/by/4.0/).
